# Formation of a Flavin-Linked Peptide

**DOI:** 10.3390/molecules19079552

**Published:** 2014-07-04

**Authors:** Masayuki Morikawa, Katsuhito Kino, Takeshi Senda, Masayo Suzuki, Takanobu Kobayashi, Hiroshi Miyazawa

**Affiliations:** Kagawa School of Pharmaceutical Sciences, Tokushima Bunri University, 1314-1 Shido, Sanuki, Kagawa 769-2193, Japan; E-Mails: s110702@stu.bunri-u.ac.jp (M.M.); s068064@stu.bunri-u.ac.jp (T.S.); s120002@stu.bunri-u.ac.jp (M.S.); kobayashit@kph.bunri-u.ac.jp (T.K.); miyazawah@kph.bunri-u.ac.jp (H.M.)

**Keywords:** flavin, peptide, cysteine, aldehyde, Aβ peptide

## Abstract

In a previous study, we showed that formylmethylflavin (FMF) can bind to cysteine. In this study, FMF was reacted with native peptides (CG and CKLVFF) containing an N-terminal cysteine. The formation of flavin-CG and flavin-CKLVFF was confirmed using HPLC and ESI-MS. Storage of flavin-CKLVFF in DMSO at −30 °C for 7 days resulted in no detectable deposition. In contrast, flavin-CKLVFF formed deposits when stored in water at −30 °C for 1 day, but no deposit was observed in the aqueous solution of flavin-CKLVFF after 7 days storage in the presence of 0.1% Triton X-100.

## 1. Introduction

Flavins are biological oxidation reagents that can photooxidize tryptophan and tyrosine [1], thereby introducing hydroxyl groups into peptides and increasing their hydrophilicity. For example, we previously suggested that the photooxidation of amyloid beta peptides (Aβ) by flavins may result in the hydroxylation of aggregated Aβ fibrils and disruption of the aggregated fibrils [2]. The suppression of aggregation is likely to inhibit Aβ toxicity, since the deposition of Aβ in the brain parenchyma and cerebro-vasculature is a critical step in the pathogenesis of Alzheimer’s disease [3,4]. Furthermore, Aβ toxicity is linked to the assembly state of the Aβ peptides [5,6,7].

A previous report showed that a short Aβ fragment (KLVFF; Aβ_16__–20_) binds full-length Aβ [8]. When the KLVFF sequence binds oxidation reagents, the product may alter the Aβ aggregation pathway and inhibit Aβ toxicity. We previously proposed that flavin-CKLVFF would likely disrupt aggregated Aβ fibrils [2].

**Scheme 1:**
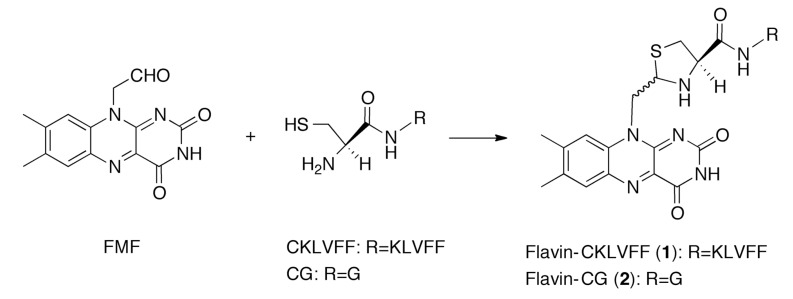
Synthesis of flavin-CKLVFF (**1**) by reaction between FMF and CKLVFF.

2-Aminoethanethiol derivatives, such as cysteine, react with aldehydes and form a five-membered heterocyclic ring via an imine [9,10,11]. Therefore, an aldehyde group is likely to react with cysteine located at the N-terminus of a peptide. Formylmethylflavin (FMF) [12,13] contains an aldehyde group and can be used to introduce a flavin into a peptide containing an N-terminal cysteine. Using this approach, we attempted the synthesis of flavin-CKLVFF. Previously, we reported the reaction between FMF and cysteine [2]. One month after our report [2], another group revealed that a flavin-linked peptide synthesized from FMF and a hydroxylamine derivative can photooxidize Aβ, and that the oxygenated Aβ exhibits decreased aggregation and cytotoxicity [14]. However, the hydroxylamine derivative used was not a native peptide. In this study, flavin-CKLVFF (**1**) was synthesized using the native peptide, CKLVFF (Scheme 1). We anticipate that flavin-CKLVFF (**1**) will exhibit decreased aggregation and cytotoxicity, comparable to that of the hydroxylamine derivative.

## 2. Results and Discussion

### 2.1. Synthesis of Flavin-CG (**2**)

**Figure 1:**
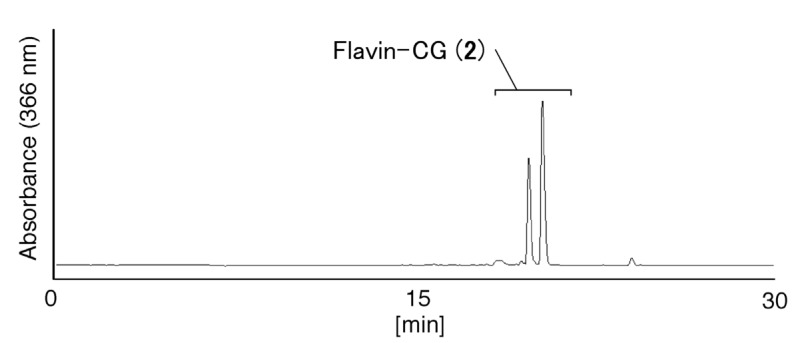
Analysis of flavin-CG (**2**). The sample was analyzed by HPLC using a Nacalai Tesque 5C18-ARII column (5 μm, 150 × 4.6 mm) eluted using a solvent of 10 mM TEAA (pH 7), 25%–35% CH_3_CN/20 min, at a flow rate of 1.0 mL/min and monitored by absorbance at 366 nm.

**Figure 2:**
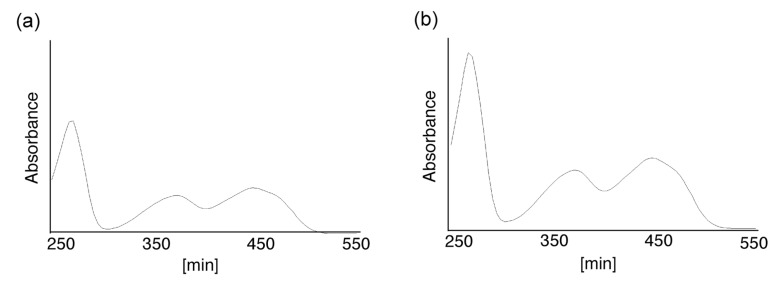
The UV-vis spectrum of flavin-CG (**2**). The UV-vis spectra of the major peaks at (**a**) 19.9 min and (**b**) 20.5 min in Figure 1 were determined.

**Figure 3:**
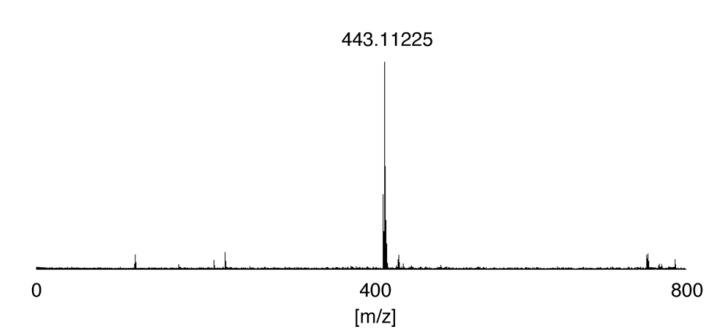
Mass spectrum of flavin-CG (**2**) obtained in negative-ion mode.

As a proof-of-concept experiment, we first determined whether a short peptide, CG, can be covalently linked to FMF. CG was reacted with FMF at 65 °C. Analysis of the reaction solution by HPLC (Figure 1) showed two major peaks, at 19.9 and 20.5 min (Figure 1). The ratio of the peak areas at 19.9 and 20.5 min in Figure 1 was determined as 35:65 and defined as the product **2a** and **2b**, respectively. Then, the UV-vis spectra of the peaks were shown in Figure 2. These peaks were isolated and analyzed using ESI-MS in negative-ion mode (Figure 3) and shown to be due to flavin-CG (**2**).

Since flavin-cysteine contains a five-membered heterocyclic ring, two diastereomers of flavin-cysteine were detected using HPLC [2]. Flavin-CG (**2**) also contains this five-membered heterocyclic ring, so the two peaks in Figure 1 are likely diastereomers of flavin-CG (**2**).

### 2.2. Synthesis of Flavin-CKLVFF (1) Using the Native Peptide

The reaction between FMF and cysteine [2] and the synthesis of flavin-CG (**2**) (Section 2.1) were performed in water. However, CKLVFF is poorly soluble in water, so to obtain high concentrations of CKLVFF, the solvent was changed to DMSO.

**Figure 4:**
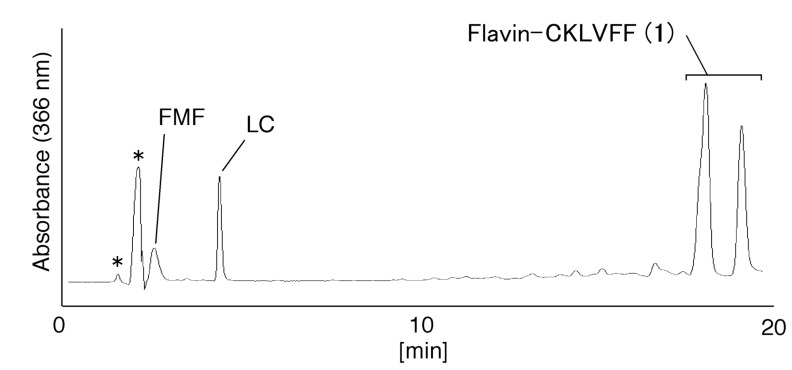
Analysis of flavin-CKLVFF (**1**). The sample was analyzed by HPLC using a Nacalai Tesque 5C18-ARII column (5 μm, 150 × 4.6 mm) and eluted using a solvent of 10 mM TEAA (pH 7), 25%–35% CH_3_CN/20 min, at a flow rate of 1.0 mL/min. The eluate was monitored by absorbance at 366 nm. “*” indicates products not formed from FMF.

**Figure 5:**
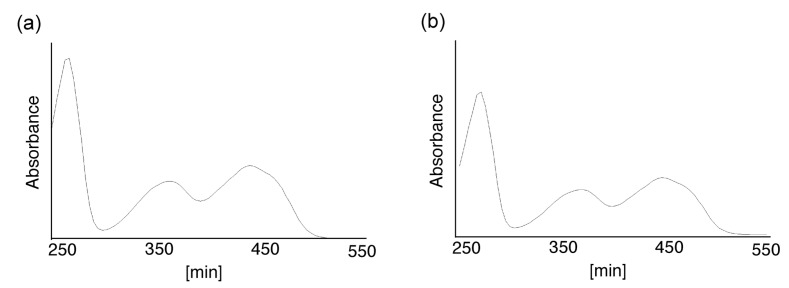
The UV-vis spectrum of flavin-CKLVFF (**1**). The UV-vis spectra of the major peaks at (**a**) 18.1 min and (**b**) 19.0 min in Figure 4 were determined.

FMF and CKLVFF in DMSO were reacted at 65 °C for 1 h, and then the solvent was replaced with water using a Sep-Pak cartridge. Analysis of the reaction solution by HPLC provided the profiles shown in Figure 4. FMF and its degradation product, lumichrome (LC), were detected at 2.5 and 4.4 min, respectively. Two major peaks were detected at 18.1 and 19.0 min. The ratio of the peak areas at 18.1 and 19.0 min in Figure 4 was determined as 60:40 and defined as the product **1a** and **1b**, respectively. Then, the UV-vis spectra of the peaks were shown in Figure 5.

**Figure 6:**
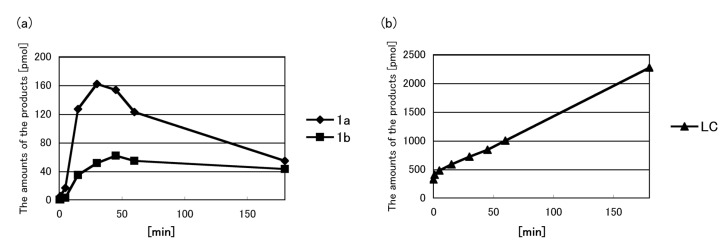
Time course analysis of the reaction between FMF and CKLVFF. FMF and CKLVFF in DMSO were reacted at 65 °C for 0–3 h, and then the solution was analyzed by HPLC using a Nacalai Tesque 5C18-ARII column (5 μm, 150 × 4.6 mm) eluted with a solvent mixture of 10 mM TEAA (pH 7), 25%–35% CH_3_CN/20 min, at a flow rate of 1.0 mL/min) and monitored by absorbance at 366 nm. The amount of product eluting at 18.1 (**1a**), 19.0 min (**1b**), and the amount of LC was determined from the area under the peak at 18.1, 19.0, and 4.4 min. **1a**, **1b** and FMF are postulated to have the same extinction coefficient. (**a**) Closed diamonds and closed squares indicate the amount of product eluting at 18.1 (**1a**) and 19.0 min (**1b**), respectively. (**b**) Closed triangles indicate the amount of LC.

Next, the time course of the reaction between FMF and CKLVFF was determined. The reaction was carried out at 65 °C for 0–3 h; samples were withdrawn periodically and analyzed using HPLC. The time-course profiles of the 18.1 and 19.0 min peaks are shown in Figure 6. The intensities of both peaks increased between 0–30 min, and then decreased after 45 min. In contrast, the amount of LC increased throughout the 3 h experiment, showing that flavin-CKLVFF (**1**) gradually degraded to LC at 65 °C.

**Figure 7:**
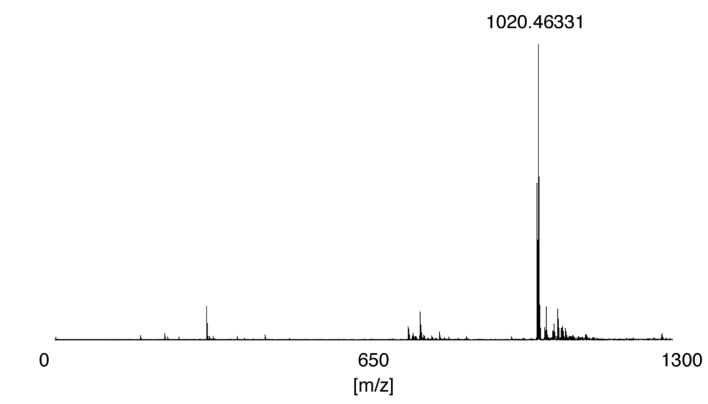
Mass spectrum of flavin-CKLVFF (**1**) obtained in negative-ion mode.

The products providing the peaks at 18.1 and 19.0 min in Figure 4 were isolated and analyzed using electrospray ionization-mass spectrometry (ESI-MS) in negative-ion mode (Figure 7). Both peaks were identified as flavin-CKLVFF (**1**). Since flavin-CKLVFF (**1**) also contains the five-membered heterocyclic ring, the peaks in Figure 4 are likely diastereomers of flavin-CKLVFF (**1**).

### 2.3. Stability of Flavin-CKLVFF (**1**)

**Figure 8:**
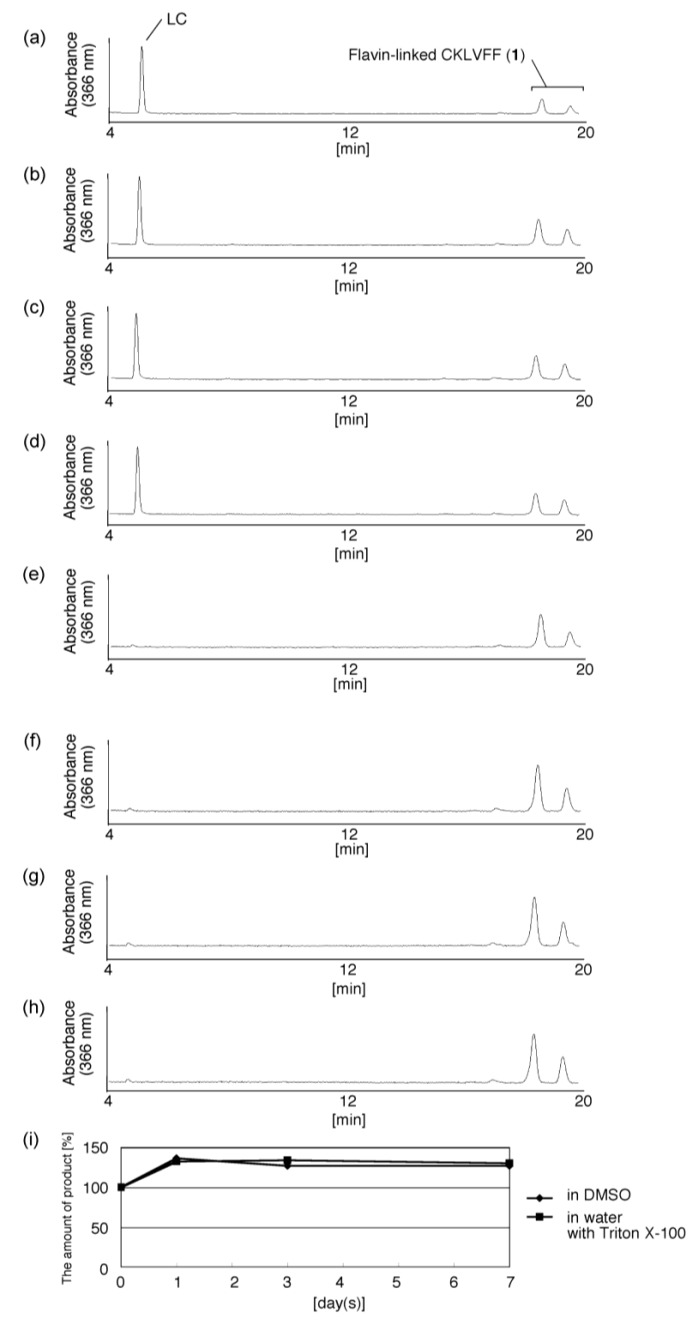
Stability of flavin-CKLVFF (**1**). FMF and CKLVFF in DMSO were reacted at 65 °C for 1 h, and then the reaction solution was left for (**a**) 0 days, (**b**) 1 day, (**c**) 3 days or (**d**) 7 days in DMSO at −30 °C. FMF and CKLVFF in DMSO were also reacted at 65 °C for 1 h, the solvent was replaced with water using a Sep-Pak cartridge, and then 0.1% Triton X-100 was added. The aqueous solution containing 0.1% Triton X-100 was left for (**e**) 0 days, (**f**) 1 day, (**g**) 3 days (**h**) 7 days at −30 °C. Then, the amounts of flavin-CKLVFF in panels (a–h) were shown in a panel (**i**). Closed diamonds and closed squares indicate the amount of product in DMSO and water with 0.1% Triton X-100, respectively. The mean values in a panel (**i**) were calculated using data from three independent experiments. The samples were analyzed by HPLC on a Nacalai Tesque 5C18-ARII column (5 μm, 150 × 4.6 mm) eluted with a solvent mixture of 10 mM TEAA (pH 7), 25%–35% CH_3_CN/20 min, at a flow rate of 1.0 mL/min and monitored by absorption at 366 nm.

To determine the stability of flavin-CKLVFF (**1**) in DMSO or water at −30 °C, FMF and CKLVFF in DMSO were reacted at 65 °C for 1 h, and then the solution was left for 1 day, 3 days or 7 days in DMSO at −30 °C and analyzed using HPLC (Figure 8a–d). The increase in the amount of LC after 1 day was less than 4%, whereas the amount of flavin-CKLVFF (**1**) after 1 day of storage was 1.4 times higher than without storage. Furthermore, no significant increase in the amount of LC was detected after 7 days, and the amount of flavin-CKLVFF (**1**) after 7 days was almost the same as after 1 day. The mechanism behind these findings remains unclear, but unreacted FMF and unreacted CKLVFF might gradually react during the first day of storage.

Next, FMF and CKLVFF in DMSO were reacted at 65 °C for 1 h, and then the solvent was replaced with water using a Sep-Pak cartridge. After 1 day in water at −30 °C, a yellowish deposit was observed in the sample. Since LC is poorly soluble in water, LC might be formed from flavin-CKLVFF (**1**) and deposited in water. However, because LC solid is green, the yellowish deposit is likely to be not LC. Then, flavin-CKLVFF (**1**) might be aggregated and deposited in water as another possible mechanism. To suppress formation of this deposit, 0.1% Triton X-100 was added to flavin-CKLVFF (**1**) in water and the mixture was left for 1 day, 3 days or 7 days, and then analyzed by HPLC (Figure 8e–h). The amount of flavin-CKLVFF (**1**) did not decrease, indicating that flavin-CKLVFF (**1**) should be stored in DMSO at −30 °C, and that it is essential that the DMSO be replaced with water prior to use. If flavin-CKLVFF (**1**) must be stored in water, 0.1% Triton X-100 should be added prior to storage at −30 °C.

## 3. Experimental Section

### 3.1. Chemicals

Riboflavin (RF) was purchased from Kishida Chemical Co., Ltd. (Osaka, Japan). DMSO, NaH_2_PO_4_, Na_2_HPO_4_, triethylamine and acetic acid were purchased from Wako Pure Chemical Industries, Ltd. (Osaka, Japan). CH_3_CN was purchased from Kanto Chemical Co., Inc. (Tokyo, Japan).Sep-Pak Plus C18 cartridges were purchased from Waters Co. (Milford, MA, USA) Triethylammonium acetate buffer (TEAA) was prepared from triethylamine and acetic acid. Phosphate buffer was prepared from NaH_2_PO_4_ and Na_2_HPO_4_. CG was purchased from Bachem AG. CKLVFF was purchased from Japan Bio Services Co., Ltd. (Saitama, Japan).Triton X-100 was purchased from Nacalai Tesque Inc. (Kyoto, Japan). FMF was prepared from RF, as previously described [12].

### 3.2. Formation of Flavin-CG

FMF (2.8 mg, 10 μmoL) was suspended in 200 mM phosphate buffer (pH 7, 1 mL), and then CG (3.6 mg, 20 μmoL) was added. The suspension was stirred at 65 °C for 1 h.

Flavin-CG (**2**) was analyzed by HPLC using a Nacalai Tesque 5C18-ARII column (5 μm, 150 × 4.6 mm) eluted with a solvent mixture of 10 mM TEAA (pH 7) at a flow rate of 1.0 mL/min, 0%–30% CH_3_CN/30 min, and monitored by absorbance at 366 nm. Flavin-CG was obtained in 89% yield based on FMF, which consists of a 35:65 mixture of **2a** and **2b**.

Flavin-CG (**2**) was purified using HPLC; the isolated flavin-CG (C_19_H_20_N_6_O_5_S) was confirmed by ESI-MS: *m/z* 443.11225 (443.11321 calculated for [M]^−^). Mass spectra were recorded on an APEX-Qe 9.4T AS (Bruker Daltonics K.K., Yokohama, Japan).

### 3.3. Formation of Flavin-CKLVFF (**1**)

FMF (10 mM) and CKLVFF (1 mM) in DMSO were stored at 65 °C for 0–3 h, providing a yellowish solution. The sample was adsorbed onto a Sep-Pak Plus C18 cartridge, and then treated with 20% CH_3_CN. The Sep-Pak cartridge was washed with 20% CH_3_CN until the eluate was no longer yellow, and then the cartridge was washed with 25% CH_3_CN until the eluate was colorless. Finally, the sample was eluted from the washed Sep-Pak cartridge with 50% CH_3_CN and dried under vacuum.

Flavin-CKLVFF (**1**) was analyzed by HPLC using a Nacalai Tesque 5C18-ARII column (5 μm, 150 × 4.6 mm) eluted with a solvent of 10 mM TEAA (pH 7), 25%–35% CH_3_CN/20 min, at a flow rate of 1.0 mL/min. The eluate was monitored by absorbance at 366 nm. Flavin-CKLVFF was obtained in 23% yield based on CKLVFF, which consists of a 60:40 mixture of **1a** and **1b**.

The isolated flavin-CKLVFF (C_52_H_67_N_11_O_9_S) was confirmed by ESI-MS: *m/z* 1020.46331 (1020.47602, calculated for [M]^−^). Mass spectra were recorded on an APEX-Qe 9.4T AS (Bruker Daltonics K.K.).

### 3.4. Stability of Flavin-CKLVFF (**1**)

FMF (10 mM) and CKLVFF (1 mM) in DMSO were stirred at 65 °C for 1 h, and then the solution was left for 1 day, 3 days or 7 days in DMSO at −30 °C and analyzed by HPLC as described in Section 3.3.

To determine the stability of flavin-CKLVFF in water, FMF (10 mM) and CKLVFF (1 mM) in DMSO were stirred at 65 °C for 1 h, and then the solvent was replaced with water using a Sep-Pak cartridge as described in Section 3.3. After the addition of 0.1% Triton X-100, the solution was left for 1 day, 3 days or 7 days at −30 °C and analyzed by HPLC as described in Section 3.3.

## 4. Conclusions

This study investigated the reaction between FMF and a native peptide with a cysteine at the N-terminus. CKLVFF and CG were shown to react with FMF; the formation of flavin-CKLVFF and flavin-CG was confirmed using HPLC and ESI-MS.

The stability of flavin-CKLVFF (**1**) in DMSO or water at −30 °C was analyzed. Flavin-CKLVFF (**1**) in DMSO at −30 °C was stable, whereas flavin-CKLVFF (**1**) formed deposits when stored in water at −30 °C. However, HPLC analysis showed that storage of flavin-CKLVFF (**1**) in water for 1 day, 3 days or 7 days at −30 °C in the presence of 0.1% Triton X-100 prevented the formation of flavin-CKLVFF (**1**) deposits.
